# MicroRNA and transcription factor co-regulatory networks and subtype classification of seminoma and non-seminoma in testicular germ cell tumors

**DOI:** 10.1038/s41598-020-57834-w

**Published:** 2020-01-21

**Authors:** Guimin Qin, Saurav Mallik, Ramkrishna Mitra, Aimin Li, Peilin Jia, Christine M. Eischen, Zhongming Zhao

**Affiliations:** 1 Center for Precision Health, School of Biomedical Informatics, The University of Texas Health Science Center at Houston, Houston, TX USA; 2 Department of Cancer Biology, Sidney Kimmel Cancer Center, Thomas Jefferson University, Philadelphia, PA USA; 3 School of Computer Science and Engineering, Xi’an University of Technology, Xi’an, Shaanxi China; 4 School of Computer Science and Technology, Xidian University, Xi’an, Shaanxi China; 5 Human Genetics Center, School of Public Health, The University of Texas Health Science Center at Houston, Houston, TX USA

**Keywords:** Computational biology and bioinformatics, Systems biology

## Abstract

Recent studies have revealed that feed-forward loops (FFLs) as regulatory motifs have synergistic roles in cellular systems and their disruption may cause diseases including cancer. FFLs may include two regulators such as transcription factors (TFs) and microRNAs (miRNAs). In this study, we extensively investigated TF and miRNA regulation pairs, their FFLs, and TF-miRNA mediated regulatory networks in two major types of testicular germ cell tumors (TGCT): seminoma (SE) and non-seminoma (NSE). Specifically, we identified differentially expressed mRNA genes and miRNAs in 103 tumors using the transcriptomic data from The Cancer Genome Atlas. Next, we determined significantly correlated TF-gene/miRNA and miRNA-gene/TF pairs with regulation direction. Subsequently, we determined 288 and 664 dysregulated TF-miRNA-gene FFLs in SE and NSE, respectively. By constructing dysregulated FFL networks, we found that many hub nodes (12 out of 30 for SE and 8 out of 32 for NSE) in the top ranked FFLs could predict subtype-classification (Random Forest classifier, average accuracy ≥90%). These hub molecules were validated by an independent dataset. Our network analysis pinpointed several SE-specific dysregulated miRNAs (miR-200c-3p, miR-25-3p, and miR-302a-3p) and genes (*EPHA2, JUN, KLF4, PLXDC2, RND3, SPI1*, and *TIMP3*) and NSE-specific dysregulated miRNAs (miR-367-3p, miR-519d-3p, and miR-96-5p) and genes (*NR2F1* and *NR2F2*). This study is the first systematic investigation of TF and miRNA regulation and their co-regulation in two major TGCT subtypes.

## Introduction

Testicular germ cell tumors (TGCT) occur most frequently in men between ages of 20 and 40^1,2^. Accordingly to histology, TGCT can be separated into two major types: seminoma (SE) and non-seminoma (NSE)^1–4^, and NSE has several subtypes. While the etiology of the two TGCT subtypes is well studied, their molecular profiles, signature genetic markers, and regulatory mechanisms have not been systematically investigated, unlike other common cancers. Such an investigation is much needed now to identify molecular signatures either common in two subtypes, or unique in subtype. The molecular signatures may be further useful for clinical implications, such as patient stratification and subtype-based or personalized treatment. Currently, there are several challenges in TGCT treatment. First, TGCT patients have a high risk of relapse with poor prognosis. Second, there are severe side effects for current chemotherapy and radiotherapy that lead to development of other pathologies. Third, since most of the patients are adolescent or young men, there is a heavy burden for the patients and families in the long run^2,3^.

During the last decade, a number of studies have been conducted to explore insights into the genetic, epigenetic, and molecular mechanisms of TGCT. For example, after collecting TGCT related genes from previous studies (e.g., *CCT6A*, *IGFBP3* and *SALL2* as novel, and *KRAS*, *MYCN*, and *TPD52* as known), Alagaratnam *et al*. analyzed the differentially expressed genes and identified a gene signature for each subtype^5^. Litchfield *et al*. performed a systematic review of the genomic features of TGCT from a timeline perspective and suggested gene biomarkers for the different stages^3^. The authors summarized 25 risk loci from previous studies and identified 19 new risk loci to TGCT by analyzing the GWAS data^6^. TGCT-related genes and miRNAs were reported from two epigenetic alteration studies^2,7^. Recently, Facchini *et al*. discussed the genetic and epigenetic events associated with TGCT, as well as the molecular mechanisms of TGCT^8^. Furthermore, integrated genomic analysis of TGCT, including expression profiles, DNA methylation, somatic copy number variation, was recently performed^4^. Among the studies, telomere length (TL) was determined to be unique in TGCT and its two types (SE and NSE)^9^. Further analysis revealed that TL elongation was dominant in NSE, while TL shortening was common in SE^10^. The TGCT type-specific molecular profiles related to TL were explored using the expression data of mRNA and microRNA (miRNA), a type of short non-coding RNA (21–22 nucleotides) that targets mRNAs^11,12^, generated by The Cancer Genome Atlas (TCGA)^10^. In that study, the authors determined that both mRNA and miRNA expression profiles could clearly distinguish these two types. TGCT-related genes and miRNAs were also reported from two epigenetic alteration studies^2,7^. Since miRNAs have a key role in post-transcriptional regulation of gene expression, it is important to further explore how genes are synergistically regulated in both types, leading to the elucidation of possible regulatory modules and mechanisms unique in NSE or SE.

Gene regulation is a basic mechanism in biological processes. It is dynamic and complex. Disruption of gene expression regulation may lead to human disease or abnormal phenotypes. Transcription factors (TFs) and miRNAs are two important types of gene expression regulators: TFs regulate gene expression at the transcriptional level by binding the promoter regions while miRNAs at the post-transcriptional level by binding the 3′ untranslated regions. Both TF and miRNA regulation can be oncogenic or tumor-suppressive^13^. Importantly, TFs and miRNAs can regulate each other. When they co-regulate a common target gene, they form a feed-forward loop (FFL). FFLs are important regulatory units, which can further form gene regulatory networks. So far, the dysregulated TF-miRNA-mediated FFLs have been found in several complex diseases, including schizophrenia^14^, glioblastoma^15^, T-cell acute lymphoblastic leukemia^16^, ovarian cancer^17^, lung cancer^18^, prostate cancer^19^, pancreatic cancer^20^, myocardial infarction^21^, colorectal cancer^22^, and dental diseases (cleft lip and cleft palate)^23,24^. Yan *et al*. proposed a method, called dChip-GemiNI, to identify common (matched) and specific TF-miRNA FFLs among five cancer types^25^. A more comprehensive TF-miRNA regulation analysis in pan-cancer data revealed 26 dysregulated FFLs in 13 cancer types, and predicted candidate genes and drug targets^26^. Zhang *et al*. determined potential active miRNA-TF-gene regulatory pathways in obesity-related inflammation using network-based methods^27^. Guo *et al*. investigated the distinct regulatory roles of TFs and miRNAs from gene regulatory network perspective, and explored the data from ENCODE (Encyclopedia of DNA Elements) and GTEx (Genotype-Tissue Expression)^28^. Furthermore, several studies introduced motif or module detection methods and performed TF-miRNA-gene regulatory network analysis^29–34^. Thus far, there has been no systematic analysis of TF and miRNA regulatory FFLs in TGCT.

Here, we compared mRNA and miRNA gene expression in NSE versus SE. We identified differentially expressed mRNAs and miRNAs by using tool Limma^35,36^. We also collected TF-gene/miRNA pairs and miRNA-gene/TF pairs. Based on these intermediate results, we formed FFLs. These FFLs were used to construct TF-miRNA-target gene regulatory network in NSE and SE, respectively. Follow-up network characteristics analysis (e.g., hub nodes for TFs, miRNAs, genes) and subtype classification analysis unveiled a subset of the FFLs that might have pathogenic potential in TGCT. Finally, these significant miRNAs and genes were evaluated using an independent dataset for TGCT. Our study is the first systematic investigation of TF and miRNA regulation as well as their co-regulation in two major TGCT subtypes, NSE and SE. The analytical approaches provided an efficient way to identify significant molecules associated with TGCT.

## Results

### Identification of differentially expressed TFs, miRNAs, and genes

**Figure 1:**
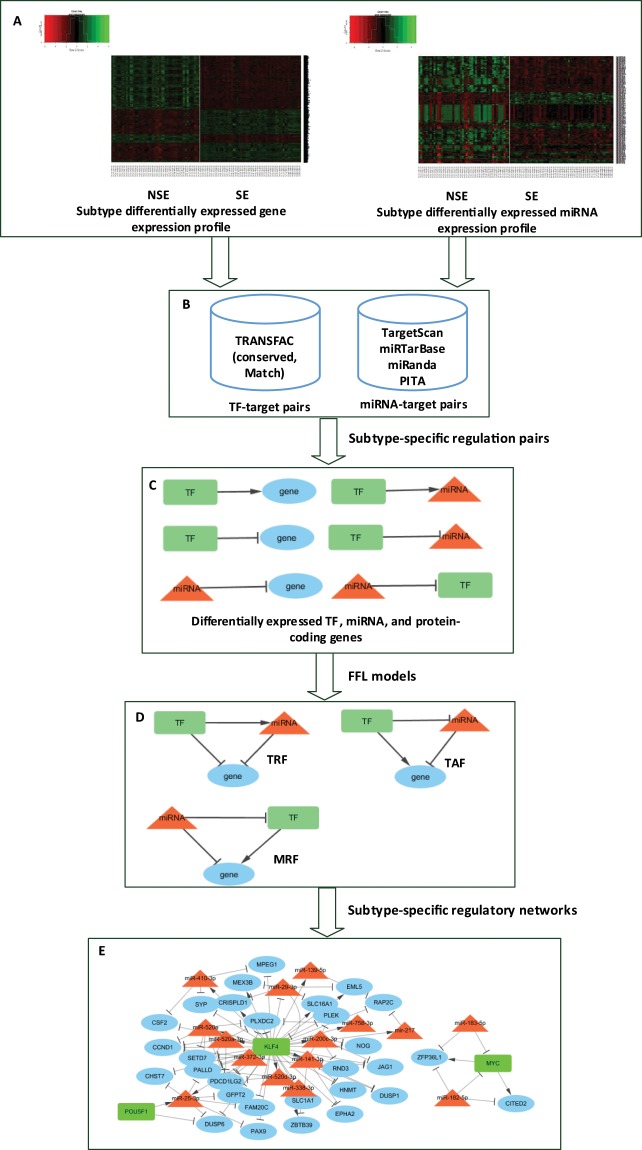
Overview of the flowchart. (**A**) mRNA and miRNA expression profiles for NSE and SE. (**B**) Predicted TF/miRNA-target pairs. (**C**) Subtype-specific regulation pairs. (**D**) Feed-forward loop (FFL) models. (**E**) Subtype-specific regulatory networks and hub detection. (Microsoft Visio 2016; RStudio version 1.1.383, https://rstudio.com/; Cytoscape version 3.7.1, https://cytoscape.org/).

Figure 1 summarizes our workflow, in which we integrated regulation pairs and gene expression profiles from different data sources, identified FFLs, and constructed and analyzed disease-specific gene regulatory networks. By following the steps described in the Materials and methods section, we identified 2,950 highly expressed genes and 167 significantly overexpressed miRNAs in NSE tumor samples (n = 48) versus SE tumor samples (n = 55) (>2 fold-change, adjusted *p*-value < 0.05). In parallel, we determined 1,969 significantly overexpressed genes and 58 highly expressed miRNAs in SE versus NSE samples (>2 fold-change, adjusted *p*-value < 0.05). In this study, we called these genes as differentially expressed genes in NSE or SE. We combined all the differentially expressed genes to identify regulatory interactions between the regulators and predicted targets.

### Regulatory interactions among TFs, miRNAs, and genes

Using the TRANSFAC data^37^ and its implemented method, MATCH^38^, we identified 56,678 TF-target gene pairs where TFs and genes were differentially expressed. We obtained 11,320 differentially expressed miRNA-target pairs using four miRNA target databases (TargetScan^39^, miRanda^40^, PITA^41^, and miRTarBase^42^). Based on these pairs, we applied Pearson's correlation coefficient (PCC) between regulators (TFs or miRNAs) and target genes, which resulted in a total of 952 FFLs with statistical significance (*p*-value < 0.05).

In NSE, we obtained 6,350 regulation pairs covering 127 unique TFs, 142 unique miRNAs, and 1,537 unique genes (Table 1). In SE versus NSE analysis, we determined 7,447 regulation pairs containing 152 unique TFs, 170 unique miRNAs, and 2,049 unique genes (Table 1). As summarized in Table 1, the activation regulation refers to the positive correlation between TFs and their targets (genes and miRNAs), while the repression regulation refers to the negative correlation between TFs and their targets, as well as between miRNAs and their targets (we only used negative regulation for miRNA-targets due to its regulation mechanism). As shown in Tables 1, 84.4% (5,362/6,350) and 93.1% (6,930/7,447) of regulation pairs were TF-target regulations for NSE-specific analysis and SE-specific analysis, respectively. Among them, ~68% were determined to be positively correlated for both NSE-specific analysis and SE-specific analysis (3,669/5,362 = 68.43%, and 4,699/6,930 = 67.81%, respectively).

**Table 1:** Summary of miRNA and TF-mediated regulations in NSE and SE.

Subtype	Regulation pair	Regulation type	# pairs	# miRNAs	# TFs	# genes
NSE	TF-gene	Activation	2,951	—	113	1,266
		Repression	1,013	—	77	576
	TF-miRNA	Activation	718	133	85	—
		Repression	680	86	52	—
	miRNA-gene	Repression	907	39	—	299
	miRNA-TF	Repression	81	17	19	—
SE	TF-gene	Activation	4,150	—	137	1,686
		Repression	1,764	—	113	1,050
	TF-miRNA	Activation	549	156	81	—
		Repression	467	125	74	—
	miRNA-gene	Repression	463	58	—	232
	miRNA-TF	Repression	54	24	34	—

Note: ‘—’ denotes no observation.

### TF-miRNA mediated feed-forward loops in SE and NSE

We identified three types of coherent FFLs in the TF-miRNA mediated gene regulatory networks (Supplementary file S1). Such coherent FFLs could reduce false positive interactions, a common issue in regulatory analysis at large-scale^43^. Specifically, they are TF repressed FFLs (TRFs), TF activated FFLs (TAFs), and miRNA repressed FFLs (MRFs). As summarized in Table 2, we identified 164 TRFs, 386 TAFs and 114 MRFs in NSE, and 86 TRFs, 163 TAFs, and 39 MRFs in SE. Although they had similar number of significant regulations (6,350 regulation pairs in NSE, and 7,447 regulation pairs in SE), the frequency of FFLs in NSE was more than twice that in SE (Hypergeometric test, *p*-value = 4.76 × 10^−53^). For example, 386 out of 664 FFLs were TAFs in NSE while for SE, 163 out of 288 FFLs were TAFs. Besides, we found only a few FFLs shared by these two subtypes, i.e. 1 shared TRF, 16 TAFs, and 5 MRFs.

**Table 2:** Summary of feed-forward loops (FFLs).

Subtype	FFL model	# FFLs	# nodes				# links		
			TFs	miRNAs	genes	TF-gene	TF-miRNA	miRNA-gene	miRNA-TF
NSE	TRF	164	17	19	55	73	69	127	—
	TAF	386	26	19	101	195	102	237	—
	MRF	114	12	14	52	58	—	105	36
SE	TRF	86	16	28	51	60	44	78	—
	TAF	163	28	26	66	118	76	116	—
	MRF	39	17	13	28	34	—	35	24

Note: abbreviations are described in main text.

We examined the number of different categories of FFLs for the top ten TFs and miRNAs (Supplementary Fig. S1). In NSE, we determined 5 TF-coding genes (*KLF4, LHX3, MAFA, NANOG*, and *POU5F1*) that had a significant role in creating TRFs, while other TF-coding genes (*ERG, FOXC1, JUN*, *NR2F1, NR2F2*, etc.) were involved in TAFs and MRFs. The top 5 TF-coding genes (*FOXC1, ERG, and NR2F2*) formed 68, 65, and 63 FFLs, respectively, accounting for 30% of the total FFLs. Among the top 10 miRNAs, miR-96-5p and miR-519d-3p were involved in 95 and 83 FFLs, accounting for 14.3% (95/664) and 12.5% (83/664) of the total number of FFLs. In SE, we identified that *SPI1, KLF4, and JUN* were in the top 3 list, which formed 59, 48, and 32 FFLs, respectively. These 3 TFs accounted for 48% of the total FFLs. For the 3 top TF-coding genes, *SPI1* was involved in 44 TAFs, and 15 TRFs, while *KLF4* formed 39 TRFs, 5 TAFs, and 4 MRFs, respectively. In these top 10 TFs of NSE and SE, there were only 2 overlapping TF-coding genes (*JUN*, and *NR2F2*). The top 10 miRNAs were miR-141-3p, miR-25-3p, miR-200c-3p, miR-29b-3p, miR-302a-3p, miR-96-5p, miR-182-5p, miR-367-3p, miR-372-3p, and miR-373-3p. The top 3 miRNAs (miR-141-3p, miR-25-3p, and miR-200c-3p) formed 32, 27, and 25 FFLs, and accounted for 11.1% (32/288), 9.4% (27/288), and 8.7% (25/288) of the total number of FFLs, respectively. In addition, among the lists of top 10 miRNAs for NSE and SE, we obtained 5 common miRNAs (miR-182-5p, miR-302a-3p, miR-367-3p, miR-373-3p, and miR-96-5p) that might have greater impact on TGCT.

### Common and subtype-specific regulatory networks

#### Topological properties of regulatory networks

We constructed miRNA and TF mediated regulatory networks in two TGCT subtypes from the identified regulation pairs (Table 1). The NSE-specific regulatory network contained 194 nodes (44 TFs, 23 miRNAs, and 127 genes) and 834 links while the SE-specific regulatory network had 168 nodes (41 TFs, 35 miRNAs, and 92 genes) and 508 links. The average degrees were 8.5 and 5.9 in these two networks, respectively. Therefore, the NSE-specific regulatory network was more strongly connected than the SE-specific regulatory network. This feature, plus more nodes and edges, indicated that NSE was more complex than SE in its regulatory mechanism. This feature might also reflect more heterogeneous samples of NSE than SE. Since the regulatory networks were directed networks, we investigated their out-degree distribution, in-degree distribution, and clustering coefficient distribution (Supplementary Fig. S2A,B). We determined that most molecules had small out-degrees and only a few genes had high out-degrees for both NSE and SE specific networks. TFs and miRNAs regulated target genes, and also regulated each other, but only a few TFs and miRNAs regulated a large number of targets. One difference between NSE and SE is that NSE had more nodes with the out-degree greater than 20. The in-degree values were more evenly distributed than those out-degree values. In addition, we determined that their average clustering coefficient distributions were similar. There were only four nodes having an average clustering coefficient greater than 0.2 for both subtypes. We searched the reported TGCT-related genes and miRNAs in related databases, including OMIM^44^, COSMIC^45^, candidate caused TGCT genes from Litchfield *et. al*.^6^, HMDD^46^, miR2Disease^47^, and PhenomiR^48^. Only 1 gene *GAB2* was regulated in our FFLs, 5 and 10 related miRNAs were found in our FFLs for NSE and SE, respectively (Supplementary file S2).

#### Hubs in the regulatory networks modulate crucial functions in TGCT tumorigenesis

Following the definition of hubs in Yu *et al*.^49^, we searched the hubs (TFs, miRNAs, and genes) in the regulatory networks by both out-degree and in-degree of nodes. We identified 32 hubs (5 TFs, 13 miRNAs, and 14 genes) and 30 hubs (8 TFs, 9 miRNAs, and 13 genes) in NSE and SE subtype-specific regulatory networks, respectively (Supplementary Table S1). There were 4 NSE-specific hub TFs (ERG, FOXC1, NR2F1, and NR2F2), 7 SE-specific hub TFs (GATA3, IRF8, KLF4, SOX9, SPI1, STAT6, and TFAP2C), as well as 1 common hub TF (JUN). Among these 5 TFs obtained from the NSE network, 2 TFs (ERG and JUN) were oncogenes, while 5 (GATA3, JUN, KLF4, SOX9, and STAT6) of the 8 hub TFs obtained from SE-specific network were oncogenes.

All of the hub miRNAs were determined from the top miRNAs. For NSE subtype, all of the 13 miRNAs belonged to four miRNA clusters: miR-183/182/96 cluster (miR-96-5p, and miR-182-5p), miR-302/367 cluster (miR-302a-3p, miR-302d-3p, and miR-367-3p), C19MC cluster (miR-519d-3p, miR-520a-3p, miR-520b, miR-520c-3p, miR-520d-3p, and miR-520e), and miR-371-3 cluster (miR-372-3p, and miR-373-3p). Of note, 9 miRNAs (miR-302a-3p, miR-302d-3p, miR-372-3p, miR-373-3p, miR-520a-3p, miR-520b, miR-520c-3p, miR-520d-3p, and miR-520e) were members of a miRNA family, miR-301/372/373/520. For the SE-specific network, the 9 hub miRNAs involved in 6 miRNA clusters: miR-106b-25/miR-17-92 clusters (miR-25-3p), miR-29 cluster (miR-29b-3p), miR-141/200c cluster (miR-141-3p, and miR-200c-3p), miR-183/182/96 cluster (miR-96-5p, and miR-182-5p), miR-302/367 cluster (miR-302a-3p, and miR-367-3p), and miR-371-3 cluster (miR-372-3p).

The miRNAs in the miR-183/182/96, miR-302/367 and miR-371-3 clusters were enriched in both NSE and SE. On the other hand, the miRNAs in C19MC cluster were enriched in the NSE type only, and the miRNAs in miR-141/200c cluster were enriched in the SE type only. We investigated the miRNAs at the cluster level. The miR-183/182/96 cluster consisted of miR-96, miR-182, and miR-183, which shared almost identical seed sequences. The miRNAs in this cluster act as oncomiRs across cancer types, including prostate, breast, and ovary cancers^50^. Furthermore, these miRNAs have an important role in regulating major cellular pathways in cancer, including apoptosis, DNA repair, metabolism, and others^50^. In our previous study, we reported that miR-96-5p and miR-183-5p were overexpressed across 12 cancer types (not including TGCT)^26^. In this work, we determined that miR-182-5p and miR-96-5p were significantly overexpressed (with fold-change 3.62 and 3.94, adjusted *p*-value 6.45 × 10^−8^ and 8.45 × 10^−10^, respectively) in the SE samples versus the NSE samples, and they were involved in 149 and 34 FFLs in NSE and SE, respectively. This observation indicated that they might have important regulatory roles in the pathology of TGCT. The miR-302/367 cluster consisted of 5 miRNAs (miR-302a, miR-302b, miR-302c, miR-302d, and miRNA-367), which were demonstrated to have vital roles in various biological processes and cellular signaling pathways^51^. The miRNAs in this cluster were activated by some TFs, including GATA6, POU5F1, NANOG, and SOX2^51^, and were related to TGCT^2,47,52^. The C19MC cluster (chromosome 19 miRNA cluster) and the miR-371-3 cluster are located on chromosome 19 and were involved in stem cell biology and tumorigenesis^53,54^. The miRNAs in the miR-371-3 cluster were biomarkers for TGCT^2,46,47^. In addition, the miR-141/200c cluster, which is part of the miR-200 family, has been reported to be associated with breast cancer^55^, whereas miR-200c-3p was found to be associated with TGCT^47^.

**Figure 2:**
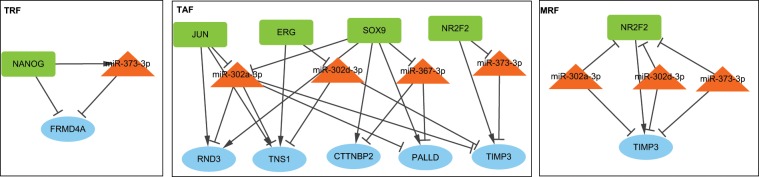
Cytoscape networks of Common FFLs in NSE and SE. (Cytoscape version 3.7.1, https://cytoscape.org/).

Eight TGCT-related miRNAs (miR-200c-3p, miR-302a-3p, miR-302c-3p, miR-302d-3p, miR-367-3p, miR-372-3p, and miR-373-3p) were hubs in both the NSE and SE-specific regulatory networks. Because we have determined the differences between the two types of TGCT, we next investigated whether there were common properties in regulation. We identified some common FFLs in NSE and SE, as shown in Fig. 2. A common TRF was NANOG—miR-373-3p—*FRMD4A*, in which NANOG was also a biomarker (and a TF) in TGCT^56^; hence this FFL might have crucial roles in TGCT tumorigenesis. There were 8 common TAFs that included 4 TFs (ERG, JUN, SOX9, and NR2F2), 4 miRNAs (miR-302a-3p, miR-302d- 3p, miR-367-3p, and miR-373-3p) and 5 target genes (*CTTNBP2, PALLD, RND3, TIMP3*, and *TNS1*). In addition, there were 3 MRFs that shared the same TF and target gene: NR2F2—miR-302a-3p/302d-3p/373-3p—*TIMP3*.

### Enrichment analysis of genes in subtype-specific regulatory networks

We conducted pathway enrichment analysis of the genes from TGCT subtype-specific regulatory networks by using Kyoto Encyclopedia of Genes and Genomes (KEGG)^57^ pathway annotations and WebGestalt tool^58^. By setting FDR (Benjamini-Hochberg adjusted *p*-value) threshold 0.05, we identified 4 oncogenic pathways that were significantly over-represented in both NSE and SE subtypes from the top 10 pathways (Table 3). Furthermore, Wnt signaling (FDR = 0.007), and Calcium signaling (FDR = 0.013) were significantly enriched in NSE subtype^57^. Of note, TGCT is male-specific cancer. For the SE subtype, we observed several relevant pathways were enriched in the context of TGCT, including Transcriptional dysregulation in cancer (FDR = 0.011), and Jak-STAT signaling (FDR = 0.017) pathways. The Jak-STAT signaling pathway is a well-known oncogenic and stemness-related pathway.

**Table 3:** Pathways enrichment analysis of the genes in subtype-specific regulatory networks by WebGestalt (FDR < 0.05).

Subtype	Pathway	Description	*p*-value	FDR	# informative genes
NSE	hsa04550	Signaling pathways regulating pluripotency of stem cells	1.11E-06	0.0002	10
	hsa05166	HTLV-I infection	1.12E-06	0.0002	13
	hsa04310	Wnt signaling pathway	7.54E-05	0.0070	8
	hsa04950	Maturity onset diabetes of the young	0.0001	0.0070	4
	hsa05200	Pathways in cancer	0.0001	0.0070	13
	hsa05205	Proteoglycans in cancer	0.0002	0.0086	9
	hsa04380	Osteoclast differentiation	0.0003	0.0130	7
	hsa05210	Colorectal cancer	0.0003	0.0130	5
	hsa04020	Calcium signaling pathway	0.0004	0.0134	8
	hsa05224	Breast cancer	0.0006	0.0170	7
	hsa04510	Focal adhesion	0.0008	0.0227	8
	hsa04022	cGMP-PKG signaling pathway	0.0013	0.0324	7
	hsa05213	Endometrial cancer	0.0017	0.0357	4
	hsa04360	Axon guidance	0.0017	0.0357	7
	hsa05215	Prostate cancer	0.0018	0.0357	5
	hsa04974	Protein digestion and absorption	0.0019	0.0357	5
	hsa05217	Basal cell carcinoma	0.0021	0.0366	4
	hsa04916	Melanogenesis	0.0031	0.0498	5
	hsa04933	AGE-RAGE signaling pathway in diabetic complications	0.0031	0.0498	5
SE	hsa04658	Th1 and Th2 cell differentiation	1.68E-06	0.0005	8
	hsa05166	HTLV-I infection	3.16E-06	0.0005	12
	hsa05200	Pathways in cancer	1.10E-05	0.0011	14
	hsa05321	Inflammatory bowel disease (IBD)	2.60E-05	0.0020	6
	hsa05161	Hepatitis B	5.14E-05	0.0031	8
	hsa05202	Transcriptional misregulation in cancer	0.0002	0.0106	8
	hsa05210	Colorectal cancer	0.0002	0.0106	5
	hsa05224	Breast cancer	0.0004	0.0135	7
	hsa05205	Proteoglycans in cancer	0.0005	0.0174	8
	hsa04630	Jak-STAT signaling pathway	0.0006	0.0174	7
	hsa04350	TGF-beta signaling pathway	0.0010	0.0274	5
	hsa04380	Osteoclast differentiation	0.0013	0.0321	6
	hsa04550	Signaling pathways regulating pluripotency of stem cells	0.0019	0.0432	6
	hsa04320	Dorso-ventral axis formation	0.0021	0.0447	3
	hsa05216	Thyroid cancer	0.0023	0.0463	3

FDR: false discovery rate.

### Regulatory features of Yamanaka factors in TGCT subtypes

Yamanaka factors include four transcription factors [KLF4, MYC, POU5F1 (OCT3/OCT4), and SOX2]. They are highly expressed in embryonic stem cells. The imbalanceness in their expression (e.g., over-expression) can induce pluripotency in both mouse and human somatic cells^59,60^. The expression of Yamanaka factors have previously been detected in testicular cancer^61^. In addition, two TF-coding genes (*POU5F1 and SOX2*) are candidate biomarkers in TGCT^56^. Their roles have been reported in testicular cancer^7,8^. This motivated us to explore the regulatory features of the Yamanaka factors in two TGCT subtypes: NSE and SE.

**Figure 3:**
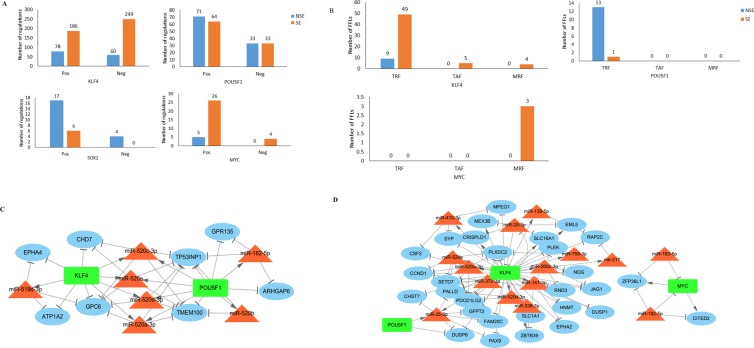
Feed forward loops (FFLs) related to Yamanaka factors. (**A**) Number of regulations of four Yamanaka factors in each TGCT subtype. (**B**) Number of FFLs in each subcategory (TRF, TAF, and MRF). (**C**) Cytoscape networks of NSE subtype-specific regulatory network. (**D**) Cytoscape networks of SE subtype-specific regulatory network. (Microsoft Excel 2013; Cytoscape version 3.7.1, https://cytoscape.org/).

First, all the four Yamanaka factors were expressed in both NSE and SE, but they had different regulatory patterns. SOX2 and MYC were up-regulated in NSE, whereas POU5F1 and KLF4 had down-regulation. Second, the number of regulation pairs of these four factors varied. KLF4 and MYC had high degree in the network (3 and 6 folds, respectively) in SE, while SOX2 had high degree (3 folds) in NSE (Fig. 3A). Finally, we explored the FFLs containing these TFs. Only POU5F1 and KLF4 formed FFLs in the NSE regulatory network; all these FFLs were TRFs, indicating that their target genes were repressed by POU5F1 and KLF4. We integrated these FFLs to construct a dense subnetwork, which consisted of 7 miRNAs, 2 TFs, 8 genes, and 41 regulations. The seven miRNAs included miR-182-5p, miR-519d-3p, and 5 miR-520a-e. For SE, we found 3 TFs (KLF4, MYC, and POU5F1) formed 52 FFLs. Similar to NSE, the majority of these FFLs (40 out of 52) were TRFs. KLF4, MYC, and POU5F1formed FFLs, which were integrated into two dense subnetworks. In the network, KLF4 positively regulated 11 miRNAs and 4 genes, but negatively regulated 2 miRNAs and 23 genes. We observed 4 of the 5 TRFs and all of the 4 MRFs constituted 4 FFLs, in which KLF4 and miR-29b-3p repressed each other and regulated their common target genes (*CRISPLD1*, *EML5*, *MEX3B*, and *SLC16A1*). MYC involved in three FFLs, MYC—miR-182-5p—*CITED2*, MYC—miR-182-5p—*ZFP36L1*, and MYC—miR-183-5p—*ZFP36L1*, all of which were MRFs. POU5F1 only formed one FFL (POU5F1—miR-25-3p—*DUSP6*), which was a TRF. Several miRNAs, including miR-141-3p, miR-182-5p, miR-183-5p, miR-25-3p, miR-519d-3p, and miR-520, as well as TGCT-related miRNAs were involved in this subnetwork (Fig. 3B–D).

### Subtype prediction based on top FFLs

For NSE and SE, we applied Random Forest classifier to each of the top 5 FFLs belonging to each FFL category to classify corresponding experimental or control class label (e.g., NSE or SE here). Using 10-fold cross-validation with 10 repeats, we obtained the classification performance on the samples for each FFL. In our experiment, the majority of the FFLs provided high classification accuracy (>= 90%) and area under the curve (AUC) (>0.9). For example, the FFL (TFAP2C—miR-520d-3p—*LYPD6*) in the category of NSE TRF produced the highest average accuracy (0.991) as well as the highest AUC (>0.999). FFL ARID5B—miR-367-3p—*STARD13* in the category of NSE MRF generated the second highest average accuracy (0.982) as well as the second highest AUC (0.999). FFL NR2F2—miR-141-3p—*EPHA2* in the category of SE TAF had the third highest average accuracy (0.979) as well as the third highest AUC (0.998). The details of average sensitivity, average specificity, average precision, average accuracy and AUC scores for the top 5 FFLs of each category are summarized in Supplementary Fig. S3, Supplementary Fig. S4, and Table 4. Since these hub genes in top 5 FFLs were important for the regulatory mechanism of TGCT, we evaluated their regulatory patterns using a validation dataset (GEO GSE99420)^62^ below.

**Table 4:** Subtype classification performance using top five FFLs of each category.

	Top five FFLs	Elements in the FFL(TF, miRNA, gene)	Avg. sensitivity	Avg. specificity	Avg. precision	Avg. accuracy
NSE TRF	FFL1	MAFA, miR-519d-3p, *MCF2L*	0.98	0.96	0.96	0.97
	FFL2	NANOG, miR-520e, *DCAF5*	0.94	0.96	0.95	0.95
	FFL3	MAFA, miR-96-5p, *ARHGAP24*	0.98	0.96	0.95	0.97
	FFL4	TFAP2C, miR-520d-3p, *LYPD6*	0.98	1.00	1.00	0.99
	FFL5	TFAP2C, miR-519d-3p, *LYPD6*	0.98	1.00	1.00	0.99
NSE TAF	FFL1	NR2F2, miR-96-5p, *ARHGAP24*	0.61	0.77	0.70	0.70
	FFL2	NR2F2, miR-96-5p, *SPATS2L*	0.63	0.75	0.68	0.69
	FFL3	NR2F1, miR-96-5p, *SPATS2L*	0.65	0.77	0.71	0.71
	FFL4	NR2F2, miR-520e, *DCAF5*	0.94	0.92	0.91	0.93
	FFL5	NR2F2, miR-520d-3p, *DCAF5*	0.95	0.91	0.90	0.93
NSE MRF	FFL1	ARID5B, miR-367-3p, *NFIX*	0.97	0.97	0.96	0.97
	FFL2	NR2F2, miR-520e, *DCAF5*	0.94	0.92	0.91	0.93
	FFL3	NR2F2, miR-520b, *DCAF5*	0.95	0.91	0.90	0.93
	FFL4	NR2F2, miR-520c-3p, *DCAF5*	0.94	0.91	0.90	0.93
	FFL5	ARID5B, miR-367-3p, *STARD13*	0.98	0.98	0.98	0.98
SE TRF	FFL1	SPI1, miR-338-3p, *ZBTB39*	0.86	0.94	0.92	0.90
	FFL2	KLF4, miR-200c-3p, *RND3*	0.88	0.96	0.95	0.92
	FFL3	SPI1, miR-142-5p, *ULK1*	0.90	0.96	0.95	0.93
	FFL4	SPI1, miR-29b-3p, *CSPG4*	0.62	0.71	0.65	0.67
	FFL5	SPI1, miR-29b-3p, *MEX3B*	0.85	0.90	0.88	0.87
SE TAF	FFL1	SPI1, miR-373-3p, *PLXDC2*	0.83	0.92	0.90	0.87
	FFL2	JUN, miR-200c-3p, *RND3*	0.89	0.96	0.96	0.93
	FFL3	SPI1, miR-141-3p, *HNMT*	0.94	0.93	0.92	0.93
	FFL4	NR2F2, miR-141-3p, *EPHA2*	0.98	0.98	0.98	0.98
	FFL5	SPI1, miR-25-3p, *FAM20C*	0.91	0.92	0.91	0.92
SE MRF	FFL1	JUN, miR-200c-3p, *RND3*	0.89	0.96	0.96	0.93
	FFL2	GATA3, miR-141-3p, *HNMT*	0.89	0.93	0.92	0.91
	FFL3	NR2F2, miR-302d-3p, *EPHA2*	0.96	0.98	0.98	0.97
	FFL4	NR2F2, miR-302a-3p, *TIMP3*	0.95	0.91	0.91	0.93
	FFL5	NR2F2, miR-302a-3p, *EPHA2*	0.97	0.96	0.96	0.97

### Subtype-specific hub regulators and targets for NSE

**Figure 4:**
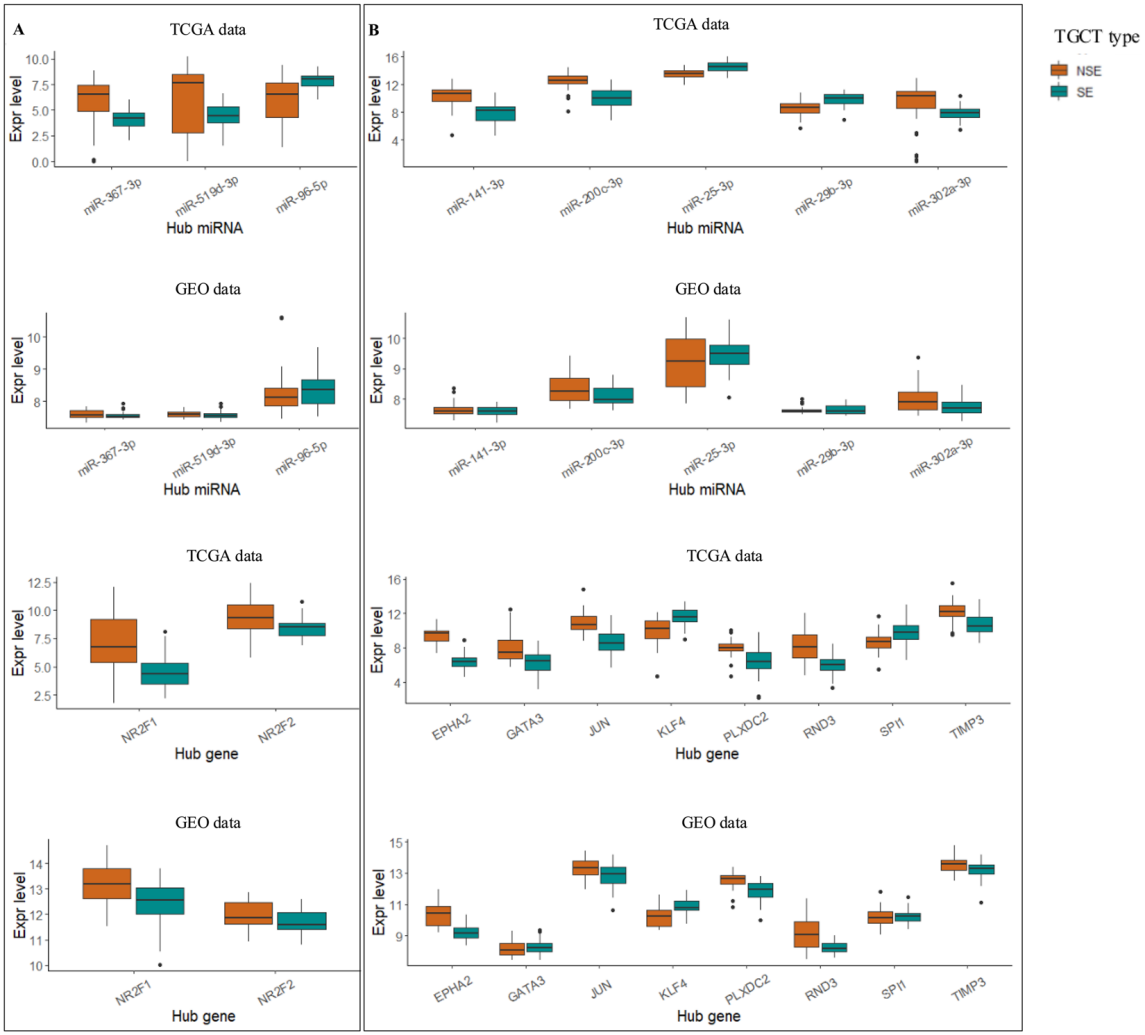
Evaluation of the hub microRNAs and genes in top FFLs by an independent dataset (GEO GSE99420). (**A**) NSE subtype. (**B**) SE subtype. On the y-axis, expression level was measured by transformed RSEM normalized count. (RStudio version 1.1.383, https://rstudio.com/).

There were 2 TFs (NR2F1 and NR2F2), 7 miRNAs (miR-367-3p, miR-519d-3p, miR-520b, miR-520c-3p, miR-520d-3p miR-520e, and miR-96-5p,) and 1 gene (*DCAF5*) identified as hubs in the top 5 FFLs that were specific for NSE. Only 2 TF-coding genes (*NR2F1* and *NR2F2*) and 3 miRNAs (miR-367-3p, miR-519d-3p, and miR-96-5p) were expressed in the GEO dataset, and their expression patterns were represented in Fig. 4. All these five genes represented the same regulatory patterns in TCGA and GEO datasets (see Supplementary Table S3). Specifically, two miRNAs (miR-96-5p, and miR-367-3p) were up-regulated, whereas the other miRNA is down-regulated. Both of the two TFs were up-regulated in NSE subtype.

### Subtype-specific hub regulators and targets for SE

For SE, there were 4 TFs (GATA3, JUN, KLF4, and SPI1), 5 miRNAs (miR-141-3p, miR-200c-3p, miR-25-3p, miR-29b-3p, and miR-302a-3p), and 4 genes (*EPHA2, PLXDC2, RND3*, and *TIMP3*) expressed in both TCGA and GEO datasets (Fig. 4 and Supplementary Table S3). Among the 5 miRNAs, 3 showed the same regulatory pattern in the two datasets, i.e., miR-200c-3p, and miR-302a-3p were down-regulated and miR-25-3p was up-regulated. In TCGA dataset, miR-141-3p was down-regulated and miR-29b-3p was up-regulated, whereas in the GEO dataset, these two miRNAs showed similar expression levels for both NSE and SE. Of note, miR-200c-3p and miR-302a-3p had stronger molecular signatures when compared to miR-25-3p in SE. Since the TFs were the top four hubs according to their out-degrees ranked from high to low score, they might play vital roles in regulating targets. *JUN* was down-regulated and *KLF4* and *SPI1* were up-regulated in both of the two datasets, even though *SPI1* was slightly up-regulated in the GEO dataset. While *GATA3* was down-regulated in TCGA dataset, it was slightly up-regulated in the GEO dataset. Hence, *JUN* and *KLF4* were likely reliable molecular signatures for SE samples. All four hub genes (*EPHA2, PLXDC2, RND3*, and *TIMP3*) were down-regulated in both datasets. By exploring the FFLs in which these hub genes were involved (Supplementary Table S2), we determined that they were regulated by several hub TFs, including *SPI1, KLF4, JUN, GATA3*, *NR2F2*, and *SOX9*. The miRNAs included miR-302a/d-3p, miR-372/373-3p, miR-520a-e, and miR141/200c, all of which have been discussed above.

### NR2F2

**Figure 5:**
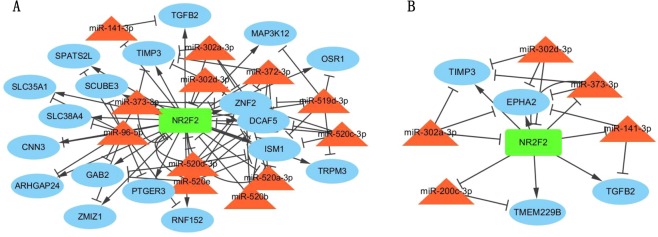
Cytoscape networks of Subtype-specific subnetwork concerning NR2F2. (**A**) NSE. (**B**) SE. (Cytoscape version 3.7.1, https://cytoscape.org/).

Interestingly, we observed that a specific TF-coding gene, *NR2F2* (nuclear receptor subfamily 2 group F member 2) was in 7 of 15 top-five FFLs (Table 4), and a key gene for classifying the NSE subtype. We investigated the FFLs in which *NR2F2* was involved, and determined that TF NR2F2 formed TAFs and MRFs with hub miRNAs (e.g., miR302-a/d-3p, miR372/373-3p, miR-519d-3p, miR520-a-e, and miR-96-5p), suggesting that it might be critical in NSE (Fig. 5A). We also determined that this TF was in 4 out of 15 top five FFLs for SE, and in 7 of 15 FFLs for NSE (Table 4). As shown in Fig. 5B, *NR2F2* and hub miRNAs (e.g. miR302-a/d-3p, miR373-3p, and miR-141/200c-3p) regulated common target genes, including two hub genes (*EPHA2* and *TIMP3*). According to the literature, *NR2F2* was overexpressed in ovarian cancer and prostate cancer, and its dysregulation was associated with testis developmental defects^63,64^, uterine fibroids^65^, and uterine implantation failure^66^. Taken together, *NR2F2* is likely a promising candidate gene in TGCT, especially in NSE.

### Common regulators and genes in two TGCT subtypes

When comparing SE-specific and NSE-specific network, we found 9 common molecules including five miRNAs (miR-182-5p, miR-302a-3p, miR-367-3p, miR-372-3p, and miR-96-5p), one TF-coding gene (*JUN*), and three non-TF genes (*FRMD4A*, *PALLD*, and *TNS1*). Of note, the out-degrees of miR-96-5p in SE- and NSE-networks were 11 and 38, respectively, whereas the in-degrees were 6 and 17, respectively. For miR-182-5p, the corresponding values of out-degrees for SE and NSE networks were 11 and 33, respectively, whereas the in-degrees were 5 and 14, respectively. The details were summarized in Supplementary Table S1. The adjusted *p*-values for these 5 miRNAs for SE and NSE analysis were 9.08 × 10^−9^, 1.69 × 10^−10^, 4.18 × 10^−7^, 2.74 × 10^−6^, and 1.38 × 10^−3^, respectively. For TF JUN, it had out-degree 29 and 26, respectively, in the SE and NSE networks, and in-degree 2 and 0, respectively. The *p*-value of JUN for SE and NSE analysis was 3.17 × 10^−14^.

## Discussion

So far, studies have been conducted to characterize the genetic, epigenetic, and molecular mechanisms of TGCT^2–4,6,7^, but not much in regulatory investigation. In this study, we first identified TGCT subtype-specific differentially expressed genes (mRNA and miRNA)^35,36^. Next, we collected TF-target gene pairs using TRANSFAC and miRNA-target gene pairs using four miRNA-target curation databases. Then, we formed FFLs by three categories: TRFs, TAFs, and MRFs. These FFLs were further used to build TF-miRNA-target gene regulatory network in two TGCT subtypes (NSE and SE). Our network analyses (such as detecting the hub nodes for TFs, miRNAs, and genes) and subtype classification analyses pinpointed subset of the FFLs that might have a significant role in the pathogenesis of TGCT subtypes. The TFs, miRNAs, and genes in the top FFLs represented promising molecular signatures in classifying TGCT types. From the dysregulated FFL networks, we assessed that most of the top FFLs could generate higher than 90% average subtype-classification accuracy through Random Forest classifier. Our study generated several SE-specific dysregulated miRNAs (miR-200c-3p, miR-25-3p, miR-302a-3p), SE-specific dysregulated genes (*EPHA2*, *JUN*, *KLF4*, *PLXDC2*, *RND3*, *SPI1*, and *TIMP3)*, NSE-specific dysregulated miRNAs (miR-367-3p, miR-519d-3p, and miR-96-5p) and NSE-specific dysregulated genes (*NR2F1* and *NR2F2)*. Furthermore, we validated the hub molecules using an independent dataset for TGCT. The validation analysis indicated that they had the similar expression patterns. Our FFL based analysis could identify distinct regulatory molecules, their interaction modules, and other features in two TGCT subtypes.

One important limitation of the study is that the dataset did not include matched control samples. This limitation was due to the original TGCT study by The Cancer Genome Atlas (TCGA), which represented the largest dataset in the field. Therefore, our results only represented the difference in expression and regulation between the two TGCT subtypes, not between TGCT tumors versus controls. Future work should include a more comprehensive understanding of the regulatory mechanisms to further uncover complex diseases like TGCT using additional multiple omics data (e.g., methylation and copy number) and regulatory relations (e.g., enhancer-gene associations). The analytical approaches proposed in this study can be applied to similar data in other cancers or complex diseases.

## Materials and Methods

### Clinical information

We downloaded TCGA generated TGCT patients’ clinical pathological information deposited in Xena database (https://xenabrowser.net/datapages, Accessed date: October 20, 2017). There was a total of 156 samples in the original clinical data file. We filtered the samples by the following two conditions as in our previous study^10^: (1) the age range of the patients was between 18 and 45; and (2) all the samples belonged to NSE or SE were verified histologically. This resulted in 48 NSE samples and 55 SE samples.

### Subtype-specific differentially expressed genes and miRNAs

Both the mRNA and miRNA expression profiles for the TGCT patient samples were downloaded from TCGA. We filtered the genes and miRNAs using the same procedure as in our previous study^10^. Briefly, for gene expression profile, we removed the genes having a log2-transformed RSEM expression level less than 1 in more than 50% of the samples^10,67^. For miRNA expression profiles, we removed the miRNAs with missing values in more than 10% of the samples, and only retained those miRNAs that had log2-transformed RSEM expression levels greater than 3.46 in more than 10% of the samples^10,68^.

Since the matched normal samples were unavailable in TCGA, we identified the differentially expressed genes and miRNAs between NSE and SE using statistical tool Limma implemented in R package^35,36^. A gene (or miRNA) was considered differentially expressed in NSE samples versus SE samples if they had at least 2-fold change with the adjusted *p*-value < 0.05. The same applied in the comparison of SE versus NSE. The analysis identified 2,950 genes and 167 miRNAs that were significantly highly expressed in NSE samples (i.e., NSE versus SE) and 1,969 genes and 58 miRNAs significantly highly expressed in SE samples (SE versus NSE).

### Transcriptional regulations of TF-gene and TF-miRNA

TRANSFAC is a comprehensive TF-target relation database^37^. We identified TF-gene pairs and TF-miRNA pairs according to the pipeline in previous studies^15,18^ using TRANSFAC data (release April 6, 2016). First, we retrieved the promoter region sequences, ranging from −1500 to +500 bp around each transcription start site (TSS) of human genes and miRNAs obtained from UCSC Table Browser^69^. We employed MATCH software^38^ to find the binding sites. We applied a pre-calculated stringent threshold to create a high-quality matrix, and we required a core score of 1.00 and a matrix score of 0.95 for each pair. Moreover, we only selected those TF-gene pairs that were conserved among human, mouse and rat.

### Post-transcriptional regulations of miRNA-gene and miRNA-TF

We selected three reliable miRNA-target prediction databases, TargetScan^39^ (release 7.1, June 2016), miRanda^40^ (release August 2010), and PITA^41^ (release Thursday, December 09, 2010). Furthermore, we regarded miRNA-target pairs from miRTarBase^42^ (release 7.0, September 15, 2017) in which the data were curated from low and high-throughput experimental procedures. We retained the pairs if they were present in at least two databases, which resulted in the identification of 170,544 miRNA-target pairs having a total of 697 unique miRNAs and a total of unique 12,507 target genes. Among them, a subset of the target genes was denoted as TFs.

### Significant transcriptional and post-transcriptional regulations

Before evaluating FFLs in regulatory networks, we defined significant regulations in our study using Pearson's correlation coefficient (PCC) threshold and corresponding *p*-value threshold (0.05). In biology, TFs may either activate or repress their target genes, and miRNAs typically repress their target genes. Accordingly, we evaluated the positive and negative correlation to determine TF-gene/miRNA pairs, but only negative correlation to determine miRNA-gene/TF pairs. By applying the threshold values above, we identified 18,431 significant regulation pairs using the data from NSE, but 7,447 significant regulation pairs in the SE subtype. Considering that PCC threshold values varied in literature and the pairs were candidate for further network analysis, we used PCC > 0.6 for NSE. This reduced the number of regulation pairs to 6,930, which is similar to SE.

### FFLs in NSE and SE

Since FFLs are directional, reflecting specific biological regulation, we define FFLs by three subcategories: TF represses FFLs (TRFs), TF activates FFLs (TAFs), and miRNA represses FFLs (MRFs). In the TRF model, a TF activates its target miRNA to repress a target gene indirectly, whereas the same TF also represses the same target gene directly. In the TAF model, a TF represses its target miRNA to repress a target gene indirectly, whereas the same TF activates the same target gene directly overcoming the effect of suppression by the target miRNA. In the MRF model, a miRNA represses its target TF to repress a target gene indirectly, whereas the same miRNA represses the same target gene directly. Of note, these three models represent biologically coherent FFLs^43^. In this study, we formed FFLs from the significantly correlated regulator-target pairs in NSE and SE, separately.

### Subtype-specific regulatory network construction and analysis

TGCT type-specific regulatory networks were constructed through integrating the identified FFLs in NSE and SE. We examined common and distinct properties between these two networks. We visualized the networks using Cytoscape, the network visualization software (version 3.7.1, https://cytoscape.org/)^70,71^. We analyzed the topological properties of the regulatory networks with Cytoscape plugin and identified hubs^49^.

### Validate of hub molecules

The expression patterns of three types of molecules (TF, miRNA and gene) identified as hubs were evaluated using an independent dataset from GEO (ID: GSE99420)^62^. The original study was to find gene signatures for relapse after 2 and 3 years of surveillance of TGCT. It had all the samples belonged to stage I, and could be divided into relapse or non-relapse, as well as NSE versus SE. The expression data was generated by Expression profiling by array platform. We used expression of 30 NSE and 30 SE samples from this dataset.

### Subtype classification based on top FFLs

To evaluate the classification ability of the resultant FFLs in terms of sample classification, we selected the top five FFLs from each category of FFL in NSE and SE subtype, individually. All the participating biomolecules (TF, miRNA and gene) belonging to each FFL were then used as features to perform two-class classification on the samples of the data using Random Forest classifier using R package caTools^72^. We utilized five measures [sensitivity, specificity, precision, accuracy, area under the receiver operating characteristic curve (AUC)] to evaluate the performance^36^. For a confusion matrix, there are basically four types in metrics: TP (True Positive), FN (False Negative), FP (False Positive) and TN (True Negative). Sensitivity denotes true positive rate, i.e., the proportion of actual positive test set tuples which are correctly classified. In other words, sensitivity is the fraction of true positives to the total number of true positives and false negatives.

1 $${\rm{Sensitivity}}=\frac{TP}{TP+FN}$$

Specificity is the true negative rate i.e., the proportion of actual negative test set tuples which are correctly classified. In other words, specificity is the fraction of true negatives to the total number of true negatives and false positives.

2 $${\rm{Specificity}}=\frac{TN}{TN+FP}$$

Accuracy is the proportion of all actual positive and negative test set tuples which are correctly classified, i.e., the fraction of the total number of true positives and true negatives to the total numbers of true positives, true negatives, false positives and false negatives.

3 $${\rm{Accuracy}}=\frac{TP+TN}{TP+TN+FP+FN}$$

Precision is the positive predictive rate, i.e., the fraction of the retrieved test tuples that are relevant. In other words, precision is the fraction of the true positives to the total number of true positives and false positives.

4 $${\rm{Precision}}=\frac{TP}{TP+FP}$$

For the experiment, we applied 10-fold cross-validation by repeating 10 times. Finally, we computed the average score of each evaluation metric.

## Supplementary information

Supporting Information.

Supporting Information.

Supporting Information.
