# Supplementary material for: MicroRNA and transcription factor co-regulatory networks and subtype classification of seminoma and non-seminoma in testicular germ cell tumors
Source: Sci Rep. 2020 Jan 21;10:852. doi: 10.1038/s41598-020-57834-w (PMC6972857; doi:10.1038/s41598-020-57834-w)
Supplement: Supplementary file 1 — Supporting Information. [file 41598_2020_57834_MOESM1_ESM.docx]

**MicroRNA and transcription factor co-regulatory networks and subtype classification of seminoma and non-seminoma in testicular germ cell tumors**

Guimin Qin^1,4,+^, Saurav Mallik^1,+^, Ramkrishna Mitra^2^, Aimin Li^1,3^, Peilin Jia^1^, Christine M. Eischen^2^, and Zhongming Zhao^1,5,*^

^1^Center for Precision Health, School of Biomedical Informatics, The University of Texas Health Science Center at Houston, Houston, TX, USA

^2^ Department of Cancer Biology, Sidney Kimmel Cancer Center, Thomas Jefferson University, Philadelphia, PA, USA

^3^ School of Computer Science and Engineering, Xi'an University of Technology, Xi’an, Shaanxi, China

^4^ School of Computer Science and Technology, Xidian University, Xi’an, Shaanxi, China

^5^Human Genetics Center, School of Public Health, The University of Texas Health Science Center at Houston, Houston, TX, USA

*Correspondence and requests for materials should be addressed to Z.Z. (email: zhongming.zhao@uth.tmc.edu)

+these authors contributed equally to this work

Figure S1. The bar plots representing the number of associated FFLs for the top ten TFs and miRNAs. (A) NSE. (B) SE. (RStudio version 1.1.383, URL: https://rstudio.com/)

Figure S2. Topological properties of subtype-specific regulatory networks. (A) NSE. (B) SE. (RStudio version 1.1.383, URL: https://rstudio.com/)

Figure S3. AUC plots of the subtype classification for the top five FFLs of each category for SE subtype. Each row represents one of the top five FFLs (FFL1, FFL2, FFL3, FFL4, and FFL5), whereas each column is a FFL category. (RStudio version 1.1.383, URL: https://rstudio.com/)

Figure S4. AUC plots of the subtype classification for the top five FFLs of each category for NSE subtype. Each row represents one of the top five FFLs (FFL1, FFL2, FFL3, FFL4, and FFL5), whereas each column is a FFL category. (RStudio version 1.1.383, URL: https://rstudio.com/)

Table S1. Hubs in subtype-specific regulatory networks

| Subtype | Hub | Out-degree | In-degree | logFC | *p*-value | adj. *p*-value |
| --- | --- | --- | --- | --- | --- | --- |
| NSE | hsa-miR-519d-3p | 42 | 17 | 1.53 | 0.001581 | 0.002641 |
|  | hsa-miR-96-5p | 38 | 17 | -1.95 | 2.26E-09 | 9.08E-09 |
|  | hsa-miR-182-5p | 33 | 14 | -2.06 | 2.93E-11 | 1.69E-10 |
|  | ERG | 32 | 3 | 1.49 | 1.01E-10 | 5.11E-10 |
|  | FOXC1 | 31 | 2 | 1.61 | 5.10E-10 | 2.32E-09 |
|  | hsa-miR-520d-3p | 29 | 17 | 2.44 | 6.30E-07 | 1.76E-06 |
|  | hsa-miR-367-3p | 28 | 10 | 1.83 | 1.38E-07 | 4.18E-07 |
|  | NR2F1 | 28 | 0 | 2.72 | 5.02E-11 | 2.65E-10 |
|  | hsa-miR-520b | 27 | 10 | 1.74 | 0.002048 | 0.003335 |
|  | hsa-miR-520a-3p | 26 | 14 | 1.3 | 0.024941 | 0.035077 |
|  | hsa-miR-373-3p | 26 | 8 | -2.1 | 5.27E-06 | 1.25E-05 |
|  | JUN | 26 | 0 | 2.27 | 3.31E-15 | 3.17E-14 |
|  | NR2F2 | 25 | 9 | 1.14 | 5.96E-06 | 1.53E-05 |
|  | hsa-miR-520e | 24 | 12 | 1.91 | 0.000393 | 0.00072 |
|  | hsa-miR-520c-3p | 22 | 8 | 1.74 | 0.002025 | 0.003308 |
|  | hsa-miR-372-3p | 22 | 6 | -2.21 | 1.01E-06 | 2.74E-06 |
|  | hsa-miR-302d-3p | 21 | 9 | 1.08 | 0.00938 | 0.013859 |
|  | hsa-miR-302a-3p | 20 | 9 | 1.44 | 0.000782 | 0.001375 |
|  | TNS1 | 0 | 18 | 1.04 | 1.04E-05 | 2.60E-05 |
|  | GPC6 | 0 | 15 | 3.33 | 2.46E-26 | 1.29E-24 |
|  | ITGB8 | 0 | 15 | 2.17 | 3.89E-12 | 2.40E-11 |
|  | TET2 | 0 | 14 | -1.44 | 1.84E-10 | 8.97E-10 |
|  | BICC1 | 0 | 13 | 1.67 | 1.78E-05 | 4.27E-05 |
|  | TP53INP1 | 0 | 13 | -1.09 | 1.69E-13 | 1.28E-12 |
|  | DCAF5 | 0 | 12 | -1.66 | 3.39E-18 | 4.99E-17 |
|  | FRMD4A | 0 | 12 | 1.07 | 1.28E-07 | 4.12E-07 |
|  | PALLD | 0 | 12 | 1.12 | 1.06E-06 | 3.01E-06 |
|  | HABP4 | 0 | 11 | -1.43 | 5.94E-19 | 9.67E-18 |
|  | KIF26B | 0 | 11 | 3.3 | 1.41E-20 | 2.93E-19 |
|  | PDCD4 | 0 | 11 | -1.06 | 1.08E-14 | 9.73E-14 |
|  | SCN2A | 0 | 11 | -1.14 | 0.001377 | 0.002477 |
|  | ZNF2 | 0 | 11 | -1.47 | 9.12E-32 | 1.20E-29 |

Table S1. Hubs in subtype-specific regulatory networks (*cont.*)

| Subtype | Hub | Out-degree | In-degree | logFC | *p*-value | adj. *p*-value |
| --- | --- | --- | --- | --- | --- | --- |
| SE | SPI1 | 61 | 0 | -1.05 | 0.000102 | 0.000218 |
|  | KLF4 | 40 | 1 | -1.66 | 1.67E-10 | 8.19E-10 |
|  | JUN | 29 | 2 | 2.27 | 3.31E-15 | 3.17E-14 |
|  | GATA3 | 19 | 1 | 1.66 | 6.37E-08 | 2.14E-07 |
|  | TFAP2C | 18 | 0 | -2.05 | 2.50E-07 | 7.74E-07 |
|  | hsa-miR-141-3p | 17 | 9 | 2.31 | 9.17E-13 | 6.99E-12 |
|  | hsa-miR-200c-3p | 16 | 10 | 2.41 | 1.21E-15 | 1.77E-14 |
|  | IRF8 | 16 | 0 | -1.01 | 5.42E-06 | 1.40E-05 |
|  | hsa-miR-302a-3p | 14 | 5 | 1.44 | 0.000782 | 0.001375 |
|  | hsa-miR-25-3p | 13 | 8 | -1.02 | 7.81E-11 | 4.23E-10 |
|  | SOX9 | 13 | 0 | 3.39 | 4.72E-19 | 7.80E-18 |
|  | hsa-miR-29b-3p | 12 | 5 | -1.2 | 3.79E-08 | 1.26E-07 |
|  | hsa-miR-367-3p | 12 | 3 | 1.83 | 1.38E-07 | 4.18E-07 |
|  | hsa-miR-96-5p | 11 | 6 | -1.95 | 2.26E-09 | 9.08E-09 |
|  | hsa-miR-182-5p | 11 | 5 | -2.06 | 2.93E-11 | 1.69E-10 |
|  | hsa-miR-372-3p | 10 | 4 | -2.21 | 1.01E-06 | 2.74E-06 |
|  | STAT6 | 10 | 0 | 1.19 | 6.76E-12 | 4.05E-11 |
|  | TNS1 | 0 | 11 | 1.04 | 1.04E-05 | 2.60E-05 |
|  | PALLD | 0 | 10 | 1.12 | 1.06E-06 | 3.01E-06 |
|  | PLXDC2 | 0 | 10 | 1.56 | 5.18E-09 | 2.03E-08 |
|  | EPHA2 | 0 | 9 | 3.14 | 2.15E-34 | 4.74E-32 |
|  | SETD7 | 0 | 9 | 1.34 | 1.48E-11 | 8.41E-11 |
|  | FRMD4A | 0 | 8 | 1.07 | 1.28E-07 | 4.12E-07 |
|  | MCOLN2 | 0 | 8 | -2.04 | 2.18E-15 | 2.14E-14 |
|  | TAGAP | 0 | 8 | -1.25 | 3.92E-07 | 1.18E-06 |
|  | TIMP3 | 0 | 8 | 1.44 | 5.06E-09 | 1.99E-08 |
|  | CHST7 | 0 | 7 | 2.55 | 6.96E-23 | 2.10E-21 |
|  | MBNL3 | 0 | 7 | 2.12 | 1.12E-14 | 1.01E-13 |
|  | RND3 | 0 | 7 | 2.49 | 4.84E-15 | 4.54E-14 |
|  | ZFP36L1 | 0 | 7 | 1.8 | 2.77E-18 | 4.14E-17 |

Table S2. Statistics of the FFLs in which hub genes were involved

| Gene | FFL category | # FFLs | FFLs | TFs | miRNAs |
| --- | --- | --- | --- | --- | --- |
| EPHA2 | TRF | 1 | KLF4 miR-141-3p EPHA2 | KLF4 | miR-141-3p |
|  | TAF | 7 | GATA3 miR-302a-3p EPHA2 JUN miR-141-3p EPHA2 JUN miR-302a-3p EPHA2 NR2F2 miR-141-3p EPHA2 NR2F2 miR-373-3p EPHA2 SPI1 miR-141-3p EPHA2 SPI1 miR-373-3p EPHA2 | GATA3  JUN  NR2F2  SPI1 | miR-302a-3p miR-141-3p  miR-373-3p |
|  | MRF | 4 | GATA3 miR-141-3p EPHA2 NR2F2 miR-302a-3p EPHA2 NR2F2 miR-302d-3p EPHA2 NR2F2 miR-373-3p EPHA2 | GATA3  NR2F2 | miR-302a-3p  miR-302d-3p miR-141-3p  miR-373-3p |
| PLXDC2 | TRF | 5 | KLF4 miR-372-3p PLXDC2  KLF4 miR-520a-3p PLXDC2 KLF4 miR-520d-3p PLXDC2 KLF4 miR-520e PLXDC2 KLF4 miR-758-3p PLXDC2 | KLF4 | miR-520a-3p  miR-520d-3p  miR-302d  miR-372-3p  miR-758-3p |
|  | TAF | 8 | GATA3 miR-302a-3p PLXDC2 GATA3 miR-520a-3p PLXDC2 SPI1 miR-372-3p PLXDC2 SPI1 miR-373-3p PLXDC2 SPI1 miR-520a-3p PLXDC2 SPI1 miR-520d-3p PLXDC2 SPI1 miR-520e PLXDC2 SPI1 miR-758-3p PLXDC2 | GATA3  SPI1 | miR-302a-3p miR-520a-3p miR-520d-3p miR-520e  miR-372-3p miR-373-3p miR-758-3p |
|  | MRF | 0 |  |  |  |
| RND3 | TRF | 2 | KLF4 miR-200c-3p RND3  KLF4 miR-200c-3p RND3 | KLF4 | miR-200c-3p |
|  | TAF | 4 | JUN miR-200c-3p RND3 JUN miR-302a-3p RND3 JUN miR-96-5p RND3 SOX9 miR-302a-3p RND3 | JUN  SOX9 | miR-200c-3p miR-302a-3p miR-96-5p |
|  | MRF | 1 | JUN miR-200c-3p RND3 | JUN | miR-200c-3p |
| TIMP3 | TRF | 0 |  |  |  |
|  | TAF | 5 | GATA3 miR-302a-3p TIMP3 NR1H3 miR-373-3p TIMP3 NR2F2 miR-373-3p TIMP3 SOX9 miR-302a-3p TIMP3 SPI1 miR-373-3p TIMP3 | GATA3 NR1H3 NR2F2  SOX9  SPI1 | miR-302a-3p miR-373-3p |
|  | MRF | 3 | NR2F2 miR-302a-3p TIMP3 NR2F2 miR-302d-3p TIMP3 NR2F2 miR-373-3p TIMP3 | NR2F2 | miR-302a-3p miR-302d-3p miR-373-3p |

Table S3. The validation of the regulatory status of the molecules

| Subtype | Hub type | # Total | # hit | # Miss | # NA |
| --- | --- | --- | --- | --- | --- |
| NSE | miRNA | 3 | 3 | 0 | 0 |
|  | gene | 2 | 2 | 0 | 0 |
| SE | miRNA | 5 | 3 | 0 | 2 |
|  | gene | 8 | 7 | 1 | 0 |

Hit means that a molecule had same regulatory status both in our analysis and the validation dataset. Miss denotes the opposite regulatory status between the two datasets. NA represents that the molecular had almost identical expression level in the validation dataset.
